# *Mycobacterium xenopi*: Evidence for Increased Rate of Clinical Isolation

**Published:** 2009-06

**Authors:** Martin H. Bluth, Ramon Vera, Jafar Razeq, Martin Kramer, Khaled I. Abu-Lawi

**Affiliations:** 1 *Department of Pathology, Wayne State University – Medicine, Detroit, MI, USA;*; 2 *Department of Infection Control, SUNY Downstate Medical Center, Brooklyn, NY, USA;*; 3 *Department of Pathology, Microbiology Laboratory, Kings County Hospital Center, Brooklyn, NY, USA*

**Keywords:** Mycobacterium, xenopi, non-tuberculous mycobacteria (NTM), HPLC

## Abstract

In light of recent reports of increased isolation of *M. xenopi*, we reviewed the number of *M. xenopi* isolates in a hospital setting over five years. A total of 133 isolates from 100 patients were reported, of these isolates, 8 were reported over the first two years, 21 isolates in the third year, 47 isolates in year four and 57 isolates in year five. The specimen sources were mainly respiratory specimens; however a few specimens were isolated from other sources. Clinical data on 12 patients with repeated isolates are presented. Patient conditions upon admission and previous medical histories are shown and compared to earlier reports. An increased awareness of the presence of this organism is necessary since the clinical presentation of patients with *M. xenopi* can be confused with tuberculosis.

## INTRODUCTION

There is a growing interest in the diagnosis of potentially pathogenic non-tuberculous Mycobacteria (NTM) (1). This interest was fueled by an increasing number of NTM cases and by the similarity between some of the NTM infections and infections by *M. tuberculosis* (2, 3). Among NTMs of particular interest is *Mycobacterium xenopi*, which has been a common isolate in Europe and Middle East (4-7). This organism is becoming increasingly isolated in certain areas of USA (4, 8, 9). *M. xenopi* is a slow-growing scotochromogenic Mycobacterium which resembles MAI when tested by biochemical reactions. However it is negative for MAI probe, grows best at 42°C, it does not grow at 25°C (4), and it has a distinct mycolic acid pattern (10).

In this paper, we report a dramatic increase in the number of *M. xenopi* isolates reported over a five year period, and review the clinical data of patients from whom multiple isolates were obtained.

## MATERIALS AND METHODS

### Data Collection and Analysis

Records of Mycobacterium culture results at Kings County Hospital Center (KCHC) were reviewed for positive *M. xenopi* cultures during a five year period after 1990. Specimen numbers were listed in Microsoft Works database. Subsequently specimen and patient information were obtained and entered in the same database. Queries were made to obtain the number of isolates and the number of patients with *M. xenopi* for each month. A Microsoft Works spreadsheet was used to tabulate the data.

### Specimen Processing

Non-sterile specimens, such as sputum and bronchial lavage, were digested and decontaminated using NaOH/NALC (N-Acetyl Cysteine) (11). The final concentrate was suspended in buffer and inoculated into Bactec 12 B medium and/or a Mycobacterium growth indicator tube and a selective 7H11 agar plate. All cultures were incubated at 37°C and screened twice during the first two weeks and once weekly after the second week. When culture was positive, an AFB stain was performed, and the isolate was identified as described below.

### Culture Identification

Positive AFB cultures were screened first for *M. tuberculosis* and *Mycobacterium avium-intracellulare* (MAI) using Gen-Probe. If both probes were negative, cultures were identified mainly by high performance liquid chromatography (HPLC), based on the method described by Butler *et al*. (10, 12). Briefly, mycolic acids were extracted from 1-2 loopfuls from colonies according to CDC standards, and the final extract of mycolic acids was suspended in 100 μL of methylene chloride and analyzed by HPLC using a C-18 Ultrasphere-X-L analytical column and a mobile phase consisting of a changing gradient. The gradient of methanol and methylene chloride began at 98% and 2% respectively, then changed to 80% and 20% for 1 minute, then changed to 35% and 65% for the following 10 minutes. Chromatograms were analyzed by Piruette software to predict the nearest match from the CDC mycolic acid library. Colonial morphology and growth rate were evaluated for conformation. Chromatogram of *M. xenopi* isolated in our laboratory (not shown) was similar for isolates identified by HPLC elsewhere (10, 12).

## RESULTS

### Frequency of NTM Isolation

In order to determine if there is an increasing trend in the isolation of NTM, we reviewed positive Mycobacterium cultures over a five year period at KCHC. A total of 12747 specimens were received in year 1, 10500 in year 2 and 8500 in year 5 (Table 1). The percentages of *Mycobacterium tuberculosis* (MTB) isolates of all specimens received, for the years one, two and five, were 8.7%, 7.5%, and 5.3%, and the percentages of *Mycobacterium avium-intracellulare* (MAI) isolates were 6.9%, 10.3%, and 10.6%, respectively. The sharpest increase was observed in NTM group other than MAI, which was 0.9% of all cultures in year one, 1.5% in year two and 3.2% in year five.

**Table 1:** Percent of Mycobacterium isolates

	**1**	**2**	**5**

Total Specimens	12747	10500	8500
Negative cultures	83.5%	80.7%	80.9%
MTB isolates	8.7%	7.5%	5.3%
MAI isolates	6.9%	10.4%	10.6%
Other NTM isolates	0.9%	1.5%	3.2%

Records of Mycobacterium cultures at Kings County Hospital were reviewed for a five year period. The total number of specimens received for each year is shown. The table also presents the percentages of negative cultures, positive *M. tuberculosis* (MTB) cultures, positive *M. avium-intracellulare* complex (MAI) cultures and other positive nontuberculous mycobacteria (NTM) for each of the years listed.

### Review of the Number of *M. xenopi* Cases

The high proportion of *M. xenopi* isolates in the NTM group prompted us to look further back to track when the number of isolates started to rise and to determine if there is any clinical significance to these isolates. During the five year period, 133 isolates from 100 patients were reported. Table 2 shows the distribution of isolates from these 100 patients in each year. A total of 3 patient were found in year one, 5 patients in year two, 21 patients in year three, 47 patients in year four and 57 patients in year five. 125 *M. xenopi* isolates were recovered from respiratory sources (sputum, bronchial lavage and bronchial biopsy), 4 isolates from urine, 1 isolate from stool and 3 isolates from body sites that are expected to be sterile.

**Table 2:** Specimen sources of *M. xenopi* isolates

	1	2	3	4	5	Total

Sputum	3	5	19	38	51	**116**
Bronchial lavage	0	0	1	2	4	**7**
Bronchial biopsy	0	0	0	1	1	**2**
CSF	0	0	1	0	0	**1**
HIP	0	0	0	1	0	**1**
Pericardium	0	0	0	1	0	**1**
Urine	0	0	0	3	1	**4**
Stool	0	0	0	1	0	**1**
**TOTAL**	**3**	**5**	**21**	**47**	**57**	**133**

*M. xenopi* positive cultures from each specimen source was recorded over a five year period. The total number of all isolates from a particular specimen source is shown (vertical), as well as the total number of isolates for each year (horizontal).

### Review of Clinical Data of 12 Patients with Repeated *M. xenopi* Isolates

In the light of our observation of an increase in *M. xenopi* isolation, we reviewed the clinical presentation of patients from which repeated isolates of *M. xenopi* were found. Although *M. xenopi* was reported in 100 patients over a five year period, only 16 patients were found to have repeated isolates or isolates from normally sterile sites. Among these 16 patients, relevant clinical data for only 12 patients was available for review. As shown in Table 3, seven patients were males with a mean age of 50 years (range, 28-76 years) and 5 patients were females with a mean age of 33 years (range, 28-40 years). *M. xenopi* was isolated from respiratory specimens in 10 patients (cases 1-10), from pericardial fluid in case 11, and from CSF in case 12.

**Table 3:** Clinical Review of 12 Patients with Frequent Isolation of *M. xenopi*

No.	Age/Sex	Symptoms	X-ray	Previous Medical Hx	No. xen. Is/No. sps. Rec.	Other AFB isolates

1	28Y/M	F, NS, PC, W	B/L RNI	pneumonia and syphilis	2/4	none
2	76Y/M	stab wound, W	L-UL cavitary lesion	PUD, dep, COPD	28/30	none
3	65Y/M	F, NS, PC, W	R-UL, L-UL ML infiltrates	pneumonia, cecal polyp COPD	2/4	none
4	41Y/M	H, NS, PC, SOB, W	B/L RNI	AIDS, PCP, salmonella sepsis Candidiasis, cocaine dependence	2/13	none
5	39Y/M	F, C, NS, W NPC, fatigue	intestinal infiltrate B/L HA	AIDS, PCP, HT, Sch, TB	3/8	M. avium
6	53Y/M	W	B/L HA	AIDS, HT, RF, RT	2/2	none
7	47Y/M	F, NPC, NS, SOB Weight loss	B/L II	AIDS, HT, PCP	2/7	M. avium
8	33Y/F	L ear discharge, F, PC, SOB, W	B/L II	AIDS, PCP, OE	2/2	none
9	30Y/F	F, PC, SOB, CP Wight loss	B/L II	AIDS, PCP, thrush	6/10	M. avium
10	28Y/F	F, NS, PC, W Weight loss	B/L LL pneumonia	syphilis, pneumonia cocaine dependence	2/3	none
11	33Y/F	F, CP, weight loss	calcified granuloma	none	1/1	none
12	40Y/F	F, HA photophobia	mediastinal adenopathy	neurosyphilis	1/2	none

Twelve patients with repeated isolates of *M. xenopi* were recorded and their charts were reviewed for relevant clinical history. Patient symptoms, on admission, are briefly listed, as are X-ray findings, previous medical history, and the number of *M. xenopi* isolates cultured from each patient in relation to specimens submitted. F, fever; C, chills; PC, productive cough; NPC, non productive cough; NS, night sweats; W, weakness; SOB, shortness of breath; H, hemoptysis; HA, headache; AIDS, aquired Immune Deficiency Syndrome; CP, chest pain; HT, hypertension; RF, renal failure; RT, renal transplant; Sch, schizophrenia; TB, tuberculosis; COPD, chronic obstructive pulmonary disease; RNI, reticulonodular infiltrates; HA, hilar adenopathy; II, interstitial infiltrate; OE, otitis externa; PCP, pneumocystis carnii pneumonia; B/L, bilateral; PUD, peptic ulcer disease; R, right; L, left; UL, upper lobe; ML, middle lobe; LL, lower lobe; No. xen. Is/No. sps. Rec, Number of M. xenopi isolates/Number of specimens received for AFB (acid fast bacilli) culture.

Nine patients were admitted with fever (cases 1, 3, 5, 7-12). Among these 9 patients, 7 patients had cough with (cases 1, 3, 5, 7, 10) or without night sweats (cases 8, 9). Other symptoms included weakness (cases 1-6, 8, 10), shortness of breath (SOB) (cases 4, 7-9), weight loss (cases 7, 9, 11), chest pain (cases 9, 11), hemoptysis (case 4), and headache (case 12).

The most common radiographic finding was an interstitial infiltrate (cases 5, 7-9). Other radiographic presentations included reticulonodular infiltrate (cases 1, 4), bilateral hilar adenopathy (cases 5, 6), upper lobe lesions (cases 2, 3), calcified granuloma (case 11), and mediastinal adenopathy (case 12).

Review of the medical history revealed that 6 patients had AIDS (cases 4-9). Five of the AIDS patients (cases 4, 5, 7-9) also had *Pneumocystis carinii* pneumonia (PCP) and 3 had concurrent isolation of other *Mycobacterium* species (cases 5, 7, 9). Two patients had chronic obstructive pulmonary disease (COPD) (cases 2, 3), three patients had syphilis (cases 1, 10, 12), and one patient had no known disease (case 11). Only one patient had a history of tuberculosis (case 5).

## DISCUSSION

Our results show an increase in the number of *M. xenopi* isolates. At our institution, only 33 isolates of *M. xenopi* were reported during the nine years before 1990 (9). Currently, we report that 3 isolates from 3 patients were found in year 1, 5 isolates from 5 patients in year 2, 21 isolates from 17 patients in year three, 47 isolates from 39 patients in year four, and 57 isolate from 36 patients in year five. Similar observations were also described in Upstate New York (8), New Jersey (4), and Ontario Canada (13).

The clinical relevance of NTM isolates is usually determined by criteria of the American Thoracic Society (ATS) for establishing an NTM disease (14). Since the patients presented in our study had underlying disease(s), it was difficult to determine the clinical relevance of *M. xenopi* isolation. However, this organism was repeatedly isolated from some of our patients. Therefore, we included patients who were deemed to have clinical infection as judged by repeated isolation of the organism (14).

Host factors reportedly associated with *M. xenopi* disease include chronic obstructive pulmonary disease (COPD), pneumoconiosis, extrapulmonary neoplastic disease (15-18), alcohol abuse (19, 20), Pott’s disease (21), arthritis (22-24), and other factors (13, 25-27). It has also been reported as a pathogen in HIV infection and in other immunocompromised states, as both pulmonary and extrapulmonary infections, although its role as a primary versus coexistent infection remains unclear (25, 28-33). Consistent with this literature, our review showed that host factors include AIDS (50%), syphilis (25%), COPD (17%) and not known in one patient (case 11).

Disease caused by *M. xenopi* is usually pulmonary (14, 32) and the presentation can closely mimic that of tuberculosis (1, 3). Symptoms of pulmonary disease caused by *M. xenopi* mainly include: productive cough, weight loss, fever, and to a lesser extent, hemoptysis, pleuritic chest pain, night sweats, and weakness (8, 13, 34). Among the 12 patients presented, 10 were suspected to have a pulmonary infection (cases 1-10). In these patients, the most common findings were weakness (80%), productive or non-productive cough (80%), fever (70%), and night sweats (60%), while fewer patients had shortness of breath (40%), weight loss (20%), chest pain (10%), and hemoptysis (10%).

Radiographically, non-tuberculous mycobacterial infection can mimic tuberculosis (21). It is characterized by multiple thin-walled cavities mostly bilateral, affecting the upper lobes, infrequent hilar retraction due to volume loss, infrequent pleural reaction adjacent to cavitation, and reticulonodular parenchymal infiltrates (36-39). Likewise, our patients with suspected pulmonary infection presented with interstitial infiltrate (50%) reticulonodular infiltrate (20%), bilateral hilar adenopathy (20%), and upper lobe lesions (20%).

*M. xenopi* has recently been isolated from water sources (40, 41) and birds are thought to be a natural host reservoir in some countries (42). However, outbreaks of *M. xenopi* have been associated with colonized hot water sources in private residence and hospitals with the route of infection most likely by the inhalation of aerosols generated during washing or showering (32, 41, 43-46). In our study, most patients presumably acquired the organism outside the hospital since only one patient had a recent prior admission to our hospital. Further epidemiologic studies are necessary to more precisely determine the source and route of infection of these organisms. Although some patients did well on anti-tuberculosis medications, there was no evidence that *M. xenopi* was the cause of the lung damage, rather than the colonization of an already damaged one.

Since *M. xenopi* can cause pulmonary symptoms similar to tuberculosis, and the AFB smear may be positive in some cases, mycobacteriology laboratories should be able to identify and report this organism to physicians in the shortest possible time. This is important so that *M.xenopi* infection can be distinguished from tuberculosis and managed accordingly, in terms of therapy and infection control plans. In our laboratory, the use of HPLC for Mycobacterium identification has substantially reduced the time required for identification of this slow growing organism.
